# Spontaneous Intra-orbital Arteriovenous Fistula: A Rare Cause of Proptosis

**DOI:** 10.7759/cureus.5984

**Published:** 2019-10-24

**Authors:** Raiko Diaz, Ricardo Rodriguez, Patricia Almeida, Gustavo Ferrer

**Affiliations:** 1 Pulmonary Medicine, Aventura Hospital and Medical Center, Aventura, USA; 2 Emergency Medicine, Aventura Hospital and Medical Center, Aventura, USA; 3 Pulmonary and Critical Care, Aventura Hospital and Medical Center, Aventura, USA

**Keywords:** orbital arteriovenous fistula, proptosis

## Abstract

A left orbital arteriovenous fistula (AVF) is diagnosed in a patient presenting with proptosis. Intra-orbital AVFs are rare according to the literature search, and therefore, the best treatment modality remains controversial. We present a case of a patient who presented with non-specific symptoms. He was diagnosed with intra-orbital AVF and underwent a trans-arterial embolization. The procedure was complicated by the central retinal artery occlusion, which is one of the most feared complications associated with this procedure. We discuss the modalities in the diagnosis of intra-orbital AVFs as well as stress the importance of an interdisciplinary approach for its timely and efficient management.

## Introduction

Intra-orbital arteriovenous fistulas (AVF) are low flow fistulas characterized by direct communication between an artery and a vein bypassing the capillary bed. They are rarely life-threatening; however, in some cases, they may present with a progression of symptoms and may require intervention. Purely intra-orbital AVFs are very rare; diagnosis may be easily missed, and treatment, when symptomatic, remains controversial.

We present a case of a patient presenting with non-specific symptoms, in which the rarity of the disease caused a challenge with diagnosis and treatment.

## Case presentation

A Hispanic male, in his sixth decade of life, with a medical history of hyperlipidemia and hypertension, presented to an ophthalmologist office with complaints of left eye swelling, redness, and bulging. The symptoms were preceded by sneezing and a runny nose three weeks prior. He denied ocular pain or changes in his vision at the time. His ocular pressure was elevated at 24 mmHg and a presumed diagnosis of glaucoma was made. He was referred to the emergency department (ED) for imaging and further management. 

In the ED, his physical examination was remarkable for left-sided proptosis and conjunctival injection. There was no restricted eye movement or elicited ocular pain. The rest of the physical examination was unremarkable, and the labs ordered, including complete blood count (CBC) and inflammatory markers (C-reactive protein and erythrocyte sedimentation rate) were unremarkable.

Imaging performed in the ED included computed tomography (CT) of the brain which was concerning for sinusitis as well as left ocular proptosis. A brain magnetic resonance imaging (MRI) was done for further evaluation which showed enhancement along the supra-ophthalmic vein reflecting possible thrombosis or pseudotumor. However, follow up imaging with magnetic resonance venography (MRV) identified an indirect left cavernous carotid fistula with no evidence of dural venous sinus or left ophthalmic vein thrombosis.

**Figure 1:**
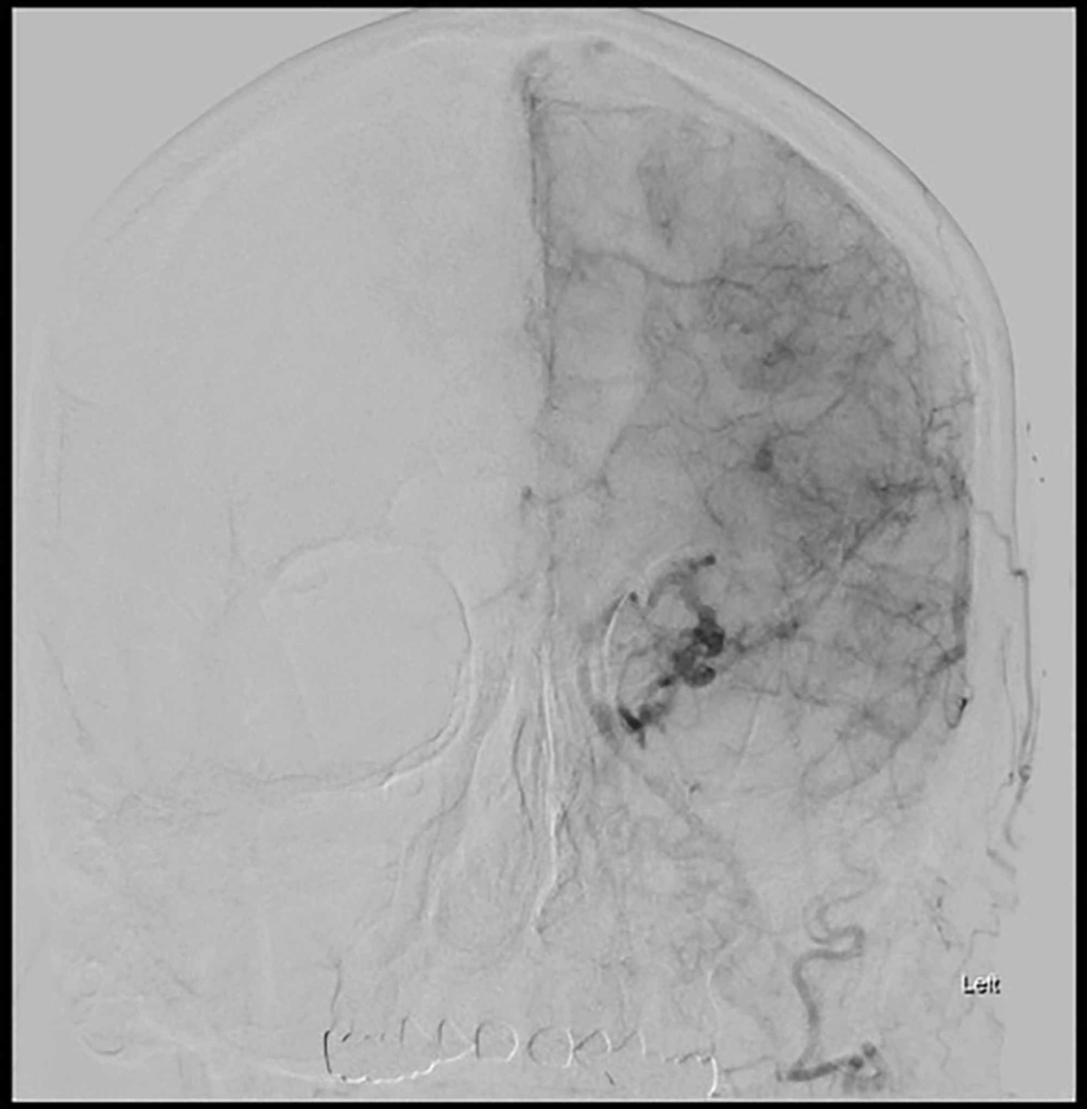
Anterior-posterior (AP) view of the left common artery carotid injection digital subtraction angiography; there is early filling of the superior ophthalmic vein, which is dilated and drains preferentially through the left facial vein

**Figure 2:**
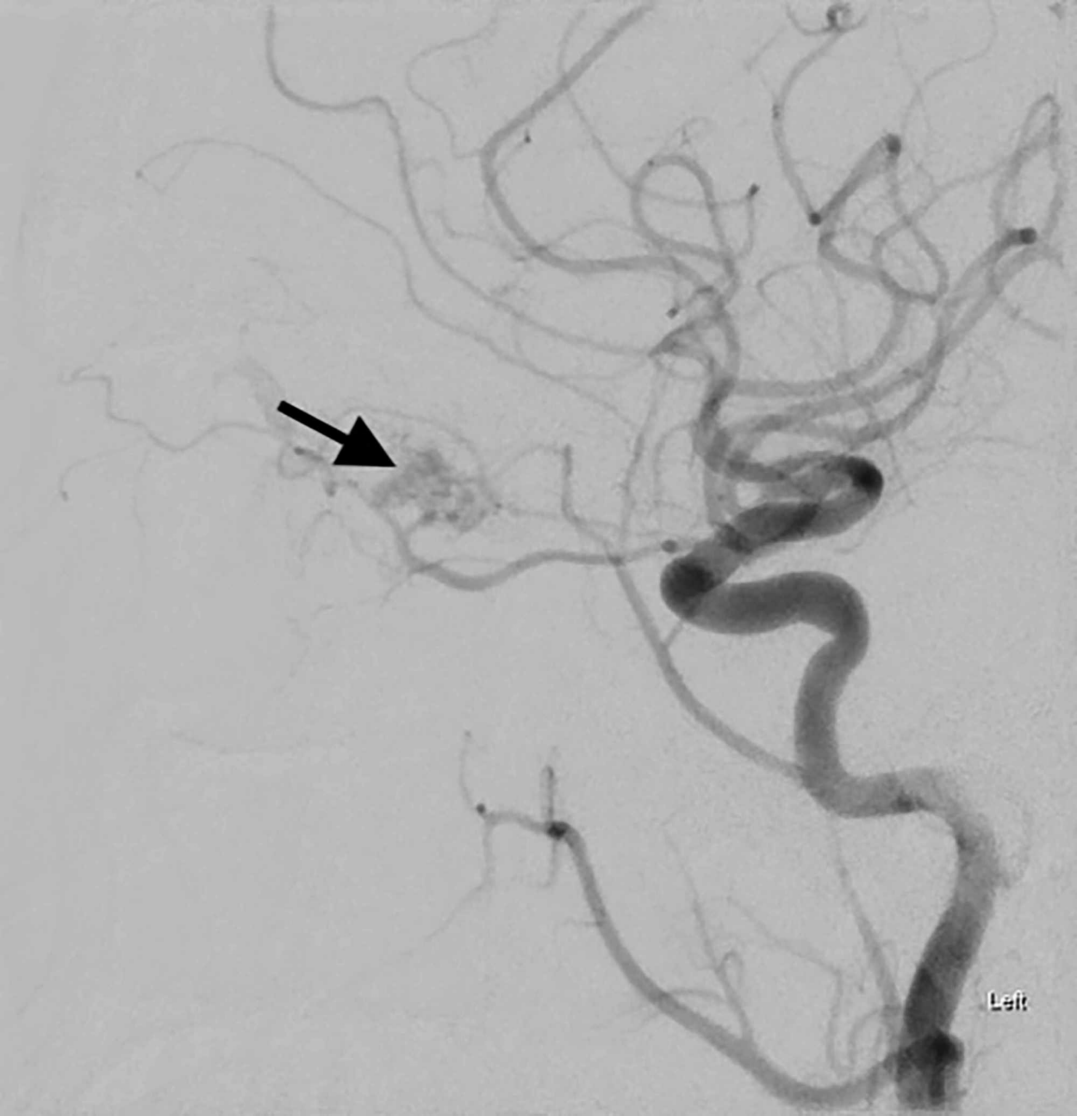
Lateral view of the left common carotid artery injection digital subtraction angiography. Early arterial phase demonstrates small branches of the left ophthalmic artery directly filling innumerable re-canalized branches of a previously thrombosed left superior ophthalmic vein

**Figure 3:**
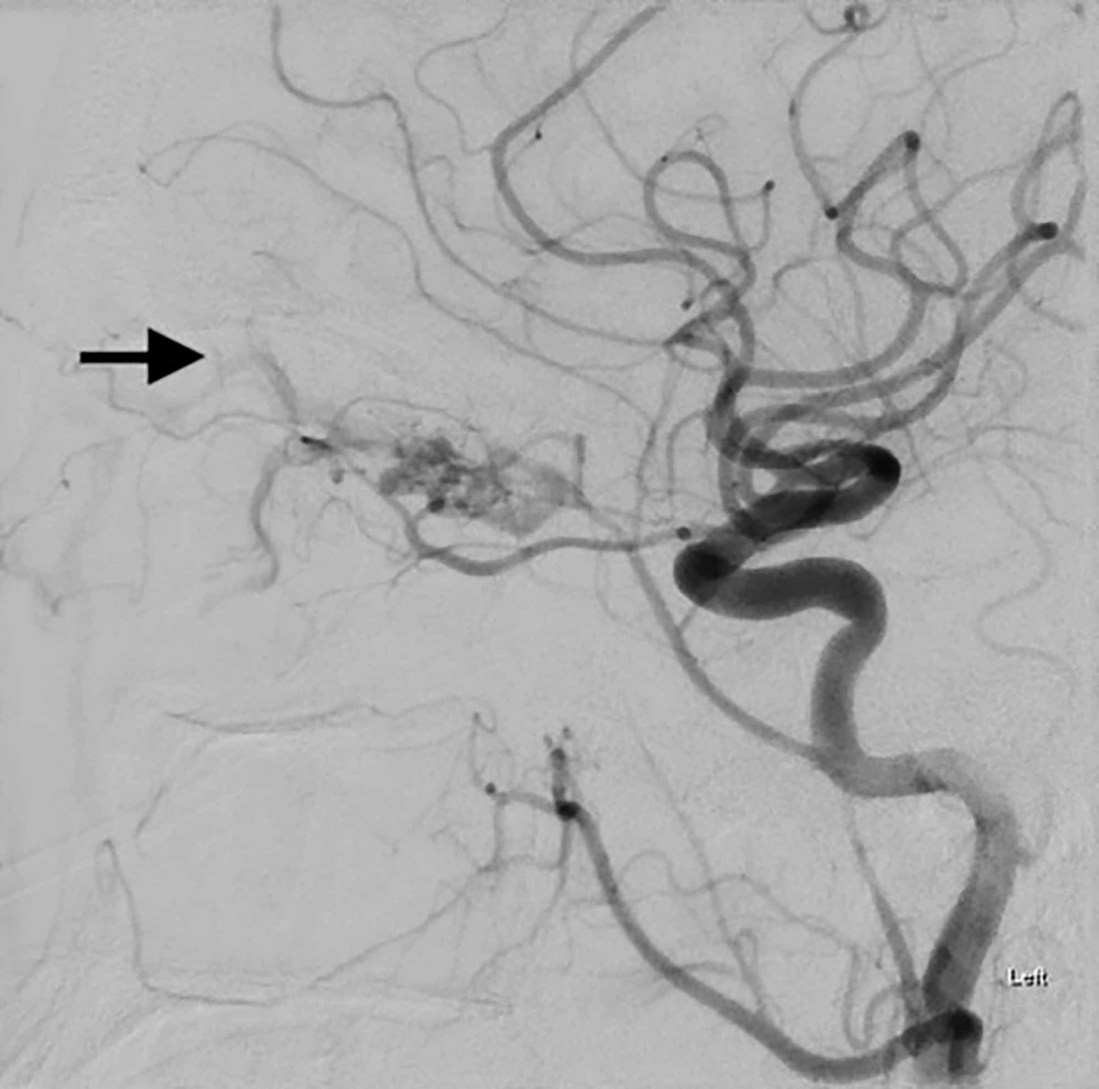
Subsequent images through the mid and delayed arterial phase demonstrate further early filling of the left ophthalmic vein, with drains anterior through the left facial vein. There is no significant drainage to the left cavernous sinus or cortical venous reflux. Therefore, this arterial venous fistula does not pose a risk of intracranial hemorrhage

The neurosurgery team was involved and a cerebral angiogram was done. The angiogram was remarkable for a left orbital AVF with a connection between the branches of the left ophthalmic artery and the left ophthalmic vein (Figures 1-3). An attempt was made to embolize or excise the fistula but was unsuccessful because the vessels in the area were small in diameter. At this time, he was referred to a local eye institute for a second opinion where conservative management was recommended due to the non-progressive nature of his symptoms.

The patient continued to have symptoms of left eye proptosis for weeks after the initial evaluation and eventually developed ocular discomfort. The decision to proceed with the intervention was made on a follow-up visit. He underwent trans-arterial embolization of the AVF. The embolization of the fistula was successful; however, the post-operative course was complicated by the loss of vision in the left eye upon awakening from the anesthesia. A fundoscopic examination was consistent with central retinal artery occlusion which led to subsequent permanent loss of vision of the left eye. 

## Discussion

AVFs in the orbit are rare. They develop from a single communication between an artery and a vein without a nidus. Most cases are secondary to traumatic events, such as a fracture of the ethmoid bone, or spontaneous. The vast majority of cases have been described in cases after trauma; however, a few cases have been documented spontaneously in patients with a prior history of hypertension, atherosclerosis, and other vascular diseases [1]. Purely intra-orbital AVF are very rare, in fact, only about 26 cases have been reported in the literature [2]. The clinical picture of intra-orbital AVFs resembles that of more common conditions, such as carotid-cavernous fistulas as well as AVMs in the orbital region [3]. The difference between an AVM and an AVF is that a fistula is a direct communication between an artery and a vein, while an AVM usually contains a bundle of arteries and veins, some of which have a connection with each other, or a nidus. In both cases, the capillary bed is bypassed and the high-pressure arterial system directly communicates with the low-pressure venous system [4].

Intra-orbital AVFs may be easily miss-diagnosed due to its rarity and common presentation. Symptoms usually involve proptosis, diplopia, ocular hypertension, and engorged episcleral vessels [1]. Vision loss and abnormal ocular movement are usually not present. The gold standard for diagnosis is angiography. Other image modalities such as MRI and MRA/MRV may be helpful for diagnostic purposes; however, in some cases, it is difficult to differentiate an intra-orbital AVF from other more common malformations, such as cavernous-carotid fistula, as was the case with our patient. The diagnosis must always be confirmed with a cerebral angiogram [5-6]. 

Carotid-cavernous fistulas are usually high-flow fistulas showing rapidly progressive symptoms. Such fistulas typically require treatment with endovascular embolization or surgical excision. Intra-orbital AVFs are typically low flow fistulas and progression of symptoms is not as dramatic. Conservative management usually includes close monitoring as well as glaucoma medications to lower the intra-ocular pressure [7]. 

Interventional methods have traditionally involved surgical excision of the fistula or trans-arterial embolization of the fistula. Trans-venous embolization is a newer modality that promises more success rates with less complications. A literature review performed by Lv et al. looked at 26 cases of documented intra-orbital AVFs in the literature who were treated using different modalities. Conservative management was used in nine patients, out of which three reported remission while six reported failure or visual deterioration. Five patients were treated using the surgical approach, out of which two reported cure and three reported failure or visual deterioration. Eleven patients were treated using the trans-arterial approach, out which one patient-reported cure and 10 reported failure or visual deterioration. The trans-venous approach was by far the most successful, with nine out of 13 patients reporting cure [2]. 

The first case of trans-venous embolization was reported in the year 2000, however, the technique was not successful until the year 2005. Since then, advances in technology and technique have progressed and it seems to carry the highest success rate. Currently, surgical excision is only performed in cases where trans-venous or trans-arterial embolization fails. The trans-arterial approach is still preferred by some physicians based on expertise. The trans-arterial approach seems to carry the highest risk of adverse events. One of the most common complications of this method is occlusion of the retinal artery as was the case for the patient presented in this case report. This occurs due to the close proximity of the arterial feeder to the central retinal artery and the small caliber of the draining branches of the superior ophthalmic vein [2].

When deciding between the trans-arterial and trans-venous approach, it is important to differentiate between an AVF and an AVM. In cases of AVM, the trans-venous approach may result in intra-orbital hemorrhage and worsening of visual function. Cerebral angiography is necessary prior to intervention to fully differentiate between the two entities. The anatomy is also very important. The trans-venous approach is much more cumbersome due to the small caliber and complex, torturous, intra-orbital venous anatomy. In comparison, the trans-arterial approach is much more direct [8].

## Conclusions

Our case report presents a rare disease which was a challenge to treat due to its rarity. Multiple treatment options exist; however, they all carry complications and are not performed very frequently. The patient in this case report could not undergo the trans-venous approach due to the small caliber and complex anatomy of his intra-orbital venous system. The trans-arterial approach was performed instead. Unfortunately, the post-operative course was complicated by central retinal artery occlusion which is one of the most common and feared complications associated with this approach. Our case demonstrates the importance of an interdisciplinary approach to the management of intra-orbital AVFs.
